# Food size and cGMP affects feeding behaviour in Pristionchus pacificus

**DOI:** 10.1186/1550-2783-8-S1-P26

**Published:** 2011-11-07

**Authors:** Silvina Kroetz, Jagan Srinivasan, Paul Sternberg, Ray L Hong

**Affiliations:** 1 Department of Biology, California State University Northridge, USA; 2 Division of Biology, California Institute of Technology, Pasadena, USA

## Background

Animals evolved different locomotory behaviors in order to find food in their environment. I studied the food seeking locomotion and pharyngeal pumping of nematodes *Pristionchus pacificus* on various food sources.

## Methods

For this study I used *P. pacificus* PS312, and the mutants *Ppa-egl-4*, which is a null mutation in the cGMP dependent protein kinase, and *Ppa-obi-1*, which is an oriental beetle pheromone insensitive mutant, and the double mutant *Ppa-egl-4;obi-1*. I tested these strains on plates containing no food and on *E.coli* OP50, HB101, *Caulobacter crescentus* (NA1000) and *Bacillus subtilis*. I analyzed locomotory behavior using an automated tracking system, and I obtained pharyngeal pumping data by visually counting with a microscope at 80X magnification.

## Results

I observed that locomotion of the strains differed on plates with no food and plates with food. On plates with no food, *P. pacificus* PS312 displayed a higher reversal rate compared to the *Ppa-obi-1* strain. The double mutant *egl-;obi-1* displayed similar locomotion patterns to *Ppa-obi-1* on HB101. Furthermore, when I compared PS312 pharyngeal pumping rates on and off food on two different size bacteria *E. coli* and *C. crescentus*, results showed a significant increased rate on PS312 on *C. crescentus*, which was the smaller bacteria.

## Conclusion

My results indicated that *Ppa-obi-1* may act in either a parallel pathway, or upstream of *Ppa-egl-4*. PS312 raised on *C. crescentus* (NA1000) for 3 generations retained memory of the food experience regardless of whether they were removed from food or placed back on NA1000 as food. Increasing bacterial size using mutant *C. crescentus* strains seem to further decrease pumping rates off food. My data suggest strong roles for food sizes and cGMP sensing proteins in maintaining feeding patterns in *P. pacificus.*

